# Prophylactic Role of Ivermectin in Severe Acute Respiratory Syndrome Coronavirus 2 Infection Among Healthcare Workers

**DOI:** 10.7759/cureus.16897

**Published:** 2021-08-05

**Authors:** Priyamadhaba Behera, Binod K Patro, Biswa M Padhy, Prasanta R Mohapatra, Shakti K Bal, Pradnya D Chandanshive, Rashmi R Mohanty, SR Ravikumar, Arvind Singh, Sudipta R Singh, Siva Santosh Kumar Pentapati, Jyolsna Nair, Gitanjali Batmanbane

**Affiliations:** 1 Community Medicine and Family Medicine, All India Institute of Medical Sciences Bhubaneswar, Bhubaneswar, IND; 2 Pharmacology, All India Institute of Medical Sciences Bhubaneswar, Bhubaneswar, IND; 3 Pulmonary Medicine and Critical Care, All India Institute of Medical Sciences Bhubaneswar, Bhubaneswar, IND; 4 General Medicine, All India Institute of Medical Sciences Bhubaneswar, Bhubaneswar, IND; 5 Forensic Medicine, All India Institute of Medical Sciences Bhubaneswar, Bhubaneswar, IND; 6 Medicine, All India Institute of Medical Sciences Bhubaneswar, Bhubaneswar, IND

**Keywords:** covid-19, ivermectin, chemoprophylaxis, healthcare workers, cohort study

## Abstract

Introduction

Healthcare workers (HCWs) are vulnerable to getting infected with severe acute respiratory syndrome coronavirus 2 (SARS-CoV-2). Preventing HCWs from getting infected is a priority to maintain healthcare services. The therapeutic and preventive role of ivermectin in coronavirus disease 2019 (COVID-19) is being investigated. Based on promising results of in vitro studies of oral ivermectin, this study was conducted with the aim to demonstrate the prophylactic role of oral ivermectin in preventing SARS-CoV-2 infection among HCWs at the All India Institute of Medical Sciences (AIIMS) Bhubaneswar.

Methods

A prospective cohort study was conducted at AIIMS Bhubaneswar, which has been providing both COVID and non-COVID care since March 2020. All employees and students of the institute who provided written informed consent participated in the study. The uptake of two doses of oral ivermectin (300 μg/kg/dose at a gap of 72 hours) was considered as exposure. The primary outcome of the study was COVID-19 infection in the following month of ivermectin consumption, diagnosed as per Government of India testing criteria (real-time reverse transcriptase polymerase chain reaction [RT-PCR]) guidelines. The log-binomial model was used to estimate adjusted relative risk (ARR), and the Kaplan-Meier failure plot was used to estimate the probability of COVID-19 infection with follow-up time.

Results

Of 3892 employees, 3532 (90.8%) participated in the study. The ivermectin uptake was 62.5% and 5.3% for two doses and single dose, respectively. Participants who took ivermectin prophylaxis had a lower risk of getting symptoms suggestive of SARS-CoV-2 infection (6% vs 15%). HCWs who had taken two doses of oral ivermectin had a significantly lower risk of contracting COVID-19 infection during the following month (ARR 0.17; 95% CI, 0.12-0.23). Females had a lower risk of contracting COVID-19 than males (ARR 0.70; 95% CI, 0.52-0.93). The absolute risk reduction of SARS-CoV-2 infection was 9.7%. Only 1.8% of the participants reported adverse events, which were mild and self-limiting.

Conclusion

Two doses of oral ivermectin (300 μg/kg/dose given 72 hours apart) as chemoprophylaxis among HCWs reduced the risk of COVID-19 infection by 83% in the following month. Safe, effective, and low-cost chemoprophylaxis has relevance in the containment of pandemic alongside vaccine.

## Introduction

The coronavirus disease 2019 (COVID-19) pandemic that started as an outbreak in Wuhan, Hubei Province of China, in December 2019 has affected around 199 million people worldwide and caused the death of approximately 4 million people [1]. In India, about 31 million people have been infected with this, and 425,195 people have died due to this disease [2]. Although healthcare workers (HCWs) represent less than 3% of the population in the large majority of countries and less than 2% in almost all low- and middle-income countries, around 14% of COVID-19 cases reported to the World Health Organization (WHO) are among HCWs, with the proportion reaching as high as 35% in some countries [3]. HCWs are more vulnerable to infection due to the very nature of their occupation, and ensuring their safety is of paramount importance in a functioning health system. Therefore, the prevention of COVID-19 disease among HCWs is a priority for all administrators and governments.

Despite the high advocacy on behavioral prophylaxis since the start of the pandemic, cases and deaths have not declined, indicating that only behavioral prophylaxis may not be enough to control the COVID-19 pandemic. In addition to behavioral prophylaxis, there is a need for an alternate safe intervention that can provide protection against COVID-19. To date, there is no effective cure available to treat [4]. The beneficial role of ivermectin in the prevention, as well as treatment of COVID-19, has been explored in the recent past [5-8]. The well-known in vitro study by Caly et al., observational studies, and an open-label randomized controlled trial (RCT) conducted so far have suggested the potential role of ivermectin as chemoprophylaxis for the prevention of COVID-19 [5-8].

This article was previously posted on the Research Square preprint platform on February 15, 2021.

## Materials and methods

The aim of this study was to demonstrate the prophylactic role of oral ivermectin in preventing severe acute respiratory syndrome coronavirus 2 (SARS-CoV-2) infection among HCWs at the All India Institute of Medical Sciences (AIIMS), Bhubaneswar, India. A prospective cohort study was conducted at the AIIMS Bhubaneswar during September to November 2020. All staff members of the institute formed the study cohort, which included the clinical staff engaged in inpatient care activities, administrative staff, and students. The protocol was approved by the Institutional Ethics Committee of AIIMS Bhubaneswar (T/IM-NF/CM&FM/20/142). All methods were performed in accordance with the relevant guidelines and regulations. Written informed consent was obtained from each participant. Efforts were taken to maintain the anonymity of the participants throughout the process. Telephonic data collection was done for the consumption of ivermectin tablets, appearance of symptoms (influenza-like illness [ILI]) and results of real-time reverse transcriptase polymerase chain reaction (RT-PCR) testing for COVID-19. The study participants were enrolled from September 17, 2020. They received ivermectin during September 20-30, 2020, and were followed up after one month of taking oral ivermectin from October 20 to October 30, 2020, to assess the outcome.

Based on a consensus statement prepared by experts from the hospital's various departments, on September 17, 2020, the prophylactic dose of oral ivermectin were made available to HCWs and students [8]. The consensus statement recommended and approved a regimen of 300 μg/kg/dose body weight with the first two doses taken 72 hours apart, followed by a once-monthly dose on the 30th day from the last dose. Ivermectin was made available free of cost to the HCWs. The intake of two doses of oral ivermectin (300 μg/kg/dose in a gap of 72 hours) was considered as exposure. HCWs were provided the oral ivermectin according to the bodyweight in the form of multiple of tablets, and dosage was similar to the doses used in the RCT by Shouman [7]. The outcome was defined as a confirmed case of COVID-19 detected by RT-PCR. HCWs were tested following the Government of India testing strategy for COVID-19 at the institute [9]. All participants who had symptoms (for ILI) or had high-risk contact with an RT-PCR-confirmed COVID-19 case were tested during the follow-up. Furthermore, the HCWs were followed up through telephonic calls to confirm their COVID-19 status after a month of distribution of ivermectin prophylaxis.

Statistical analysis was done using Stata 13.0 software (StataCorp, College Station, TX). The means and standard deviations were reported for continuous variables and proportions for categorical variables. The log-binomial model was used to estimate adjusted relative risk (ARR) [10]. Relative risk was adjusted for age, gender and profession. We also performed a sensitivity analysis, excluding those who were COVID-19 positive before the ivermectin prophylaxis. The Kaplan-Meier failure plot was used to estimate the probability of SARS-CoV-2 infection with follow-up time. COVID-19-positive HCWs and students during the study period were treated at the institute.

## Results

The institute was functioning with 3892 members during September 2020. Out of 3892, 262 were excluded from the study as they did not consent to participate in the study. Another 98 participants could not be followed up and were excluded from the study. A total of 3532 participants were included in the study. The mean (SD) age was 30.6 (8.6) years. Over half of the study participants were less than 30 years of age (53.4%), while one-third (32.3%) were in the 30- to 39-year age group. The majority of participants were male (67.6%). Approximately three-fourths (72.7%) of the participants were involved in the direct management of COVID-19 patients. Administrative staff and students comprised 13.9% and 13.4%, respectively. Among the 2567 participants, who were involved in COVID-19 patient care, 812 were doctors, 717 were nursing officers, and 1038 were supporting staff.

Uptake of ivermectin was 67.5% (62.2% two doses and 5.3% single dose). Rest of the 1147 (32.5%) participants did not consume ivermectin as prophylaxis. The symptoms suggestive of SARS-CoV-2 infection (as per WHO guideline) were present among 331 (9.4%) participants during the one-month follow-up [11]. A total of 201 (5.7%) persons within our cohort tested COVID-19 positive during the one-month follow-up period (Table 1).

**Table 1:** Characteristic features of participants (n=3532) COVID-19, coronavirus disease 2019; SARS-CoV-2, severe acute respiratory syndrome coronavirus 2

Characteristic	Number of participants, n (%)
Age (years)	
<30	1887 (53.4)
30-39	1139 (32.3)
40-49	358 (10.1)
≥50	148 (4.2)
Gender	
Male	2389 (67.6)
Female	1143 (32.4)
Profession	
Staff involved in COVID-19 patient care	2567 (72.7)
Administrative staff	492 (13.9)
Students	473 (13.4)
Ivermectin prophylaxis	
No ivermectin prophylaxis	1147 (32.5)
Received single-dose ivermectin prophylaxis	186 (5.3)
Received double-dose ivermectin prophylaxis	2199 (62.2)
Symptoms suggestive of SARS-CoV-2 infection during follow-up	
Present	331 (9.4)
Absent	3201 (90.6)
Follow-up confirmation of COVID-19 by RT-PCR	
Positive	201 (5.7)
Negative	3331 (94.3)

Ivermectin prophylaxis uptake was better with increasing age and among males. Out of 331 participants, who had symptoms suggestive of SARS-CoV-2 infection, 200 (60.4%) participants were from the group who had not taken ivermectin prophylaxis. The participants who took ivermectin prophylaxis had a lower risk (6% vs 15%) of getting symptoms suggestive of SARS-CoV-2 infection (Table 2).

**Table 2:** Distribution of participants with ivermectin prophylaxis COVID-19, coronavirus disease 2019; SARS-CoV-2, severe acute respiratory syndrome coronavirus 2

	Ivermectin two-dose prophylaxis		
Variables	Yes	No	p-value
Age (years)			<0.001
<30	1115 (50.7)	772 (57.9)	
30-39	705 (32.1)	434 (32.6)	
40-49	262 (11.9)	96 (7.2)	
≥50	117 (5.3)	31 (2.3)	
Gender			<0.001
Male	1622 (67.9)	767 (32.1)	
Female	577 (50.5)	566 (49.5)	
Profession			0.236
Staff involved in COVID-19 patient care	1582 (71.9)	985 (73.9)	
Administrative staff	306 (13.9)	186 (13.9)	
Students	311 (14.2)	162 (12.2)	
Symptoms suggestive of SARS-CoV-2 infection during follow-up			<0.001
Positive	131 (6.0)	200 (15.0)	<0.001
Negative	2068 (94.0)	1133 (85.0)	

The incidence of SARS-CoV-2 infection was found to be lower in the ivermectin prophylaxis group compared to the group without ivermectin (2.0% vs 11.7%). The absolute risk reduction was 9.7%. Participants who had taken two doses of ivermectin prophylaxis had a lower risk of contracting COVID-19 disease (RR 0.18; 95% CI, 0.13-0.25) in the following month after receiving prophylaxis. On adjusting for age, sex, and profession, the single dose of ivermectin intake was not significant for lowering the risk of COVID-19 disease (ARR 1.04; 95% CI, 0.69-1.58). However, two doses of ivermectin prophylaxis had a significantly lower risk (ARR 0.17; 95% CI, 0.12-0.23). Females had a lower risk (ARR 0.70; 95% CI, 0.52-0.93) of contracting COVID-19 disease compared to males (Table 3).

**Table 3:** Risk factors for SARS-CoV-2 infection *Adjusted for age, gender and profession. RR, relative risk; COVID-19, coronavirus disease 2019; SARS-CoV-2, severe acute respiratory syndrome coronavirus 2

Variables	Total participants	Follow-up (one month), COVID-19 positive	Unadjusted RR (95% CI)	p-value	Adjusted RR* (95% CI)	p-value
Age (years)						
<30	1887 (53.4)	116 (57.7)	Reference		Reference	
30-39	1139 (32.3)	61 (30.3)	0.87 (0.64-1.18)	0.370	0.87 (0.65-2.18)	0.392
40-49	358 (10.1)	18 (9.0)	0.82 (0.50-1.33)	0.415	0.95 (0.59-1.54)	0.848
≥50	148 (4.2)	6 (3.0)	0.66 (0.30-1.47)	0.310	0.85 (0.39-1.89)	0.694
Gender						
Male	2389 (67.6)	138 (68.7)	Reference		Reference	
Female	1143(32.4)	63 (31.3)	0.95 (0.71-1.27)	0.751	0.70 (0.52-0.93)	0.015
Profession						
Staff involved in COVID-19 patient care	2567 (72.7)	150 (74.6)	Reference		Reference	
Administrative staff	492 (13.9)	24 (11.9)	0.83 (0.55-1.27)	0.399	0.82 (0.54-1.24)	0.354
Students	473 (13.4)	27 (13.5)	0.98 (0.66-1.45)	0.908	1.09 (0.74-1.61)	0.652
Ivermectin prophylaxis						
No ivermectin prophylaxis	1147 (32.5)	133 (66.2)	Reference		Reference	
Received single-dose ivermectin prophylaxis	186 (5.3)	23 (11.4)	1.07 (0.70-1.61)	0.761	1.04 (0.69-1.58)	0.846
Received two-dose ivermectin prophylaxis	2199 (62.2)	45 (22.4)	0.18 (0.13-0.25)	<0.001	0.17 (0.12-0.23)	<0.001

**Figure 1:**
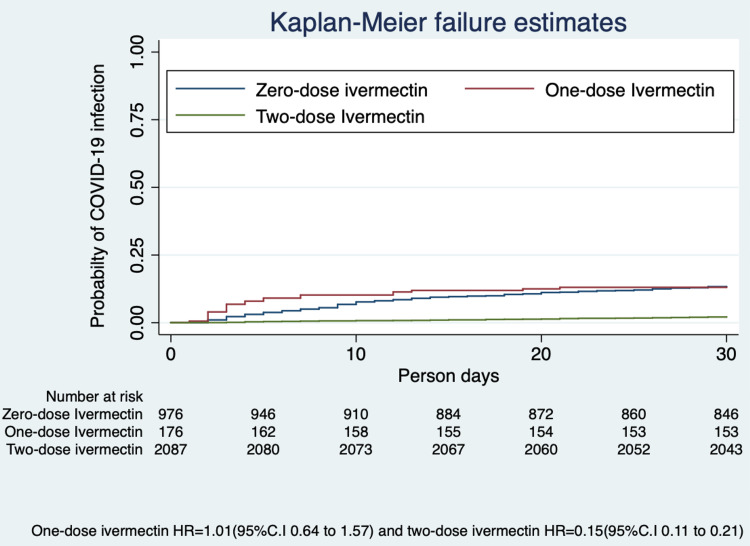
Probability of SARS-CoV-2 infection with ivermectin prophylaxis COVID-19, coronavirus disease 2019; SARS-CoV-2, severe acute respiratory syndrome coronavirus 2

We estimated the hazard ratio (HR), excluding those who had been diagnosed as COVID-19 positive before the commencement of the study using the Kaplan-Meier method. The probability of SARS-CoV-2 infection was 85% lower (HR 0.15; 95% CI, 0.11-0.21) in those taking two-dose ivermectin at the end of 30 days (Figure 1).

The information regarding adverse effects was collected through the existing pharmacovigilance services of the institute, and telephonic follow-up. A total of 42 (1.8%) participants reported adverse events following oral ivermectin. All adverse effects were self-limiting and mild in nature, and none required medication or hospitalization. Adverse events were headache, diarrhoea, nausea, itching, rashes, fatigue, vomiting, dizziness, and abdominal pain (Table 4).

**Table 4:** Adverse events following ivermectin prophylaxis (n=2385)

Adverse event	n (%)
Itching	5 (0.2)
Headache	9 (0.4)
Rashes	3 (0.1)
Diarrhoea	7 (0.3)
Dizziness	2 (0.1)
Nausea	7 (0.3)
Vomiting	3 (0.1)
Fatigue	4 (0.2)
Abdominal pain	2 (0.1)
Total	42 (1.8)

## Discussion

**Figure 2:**
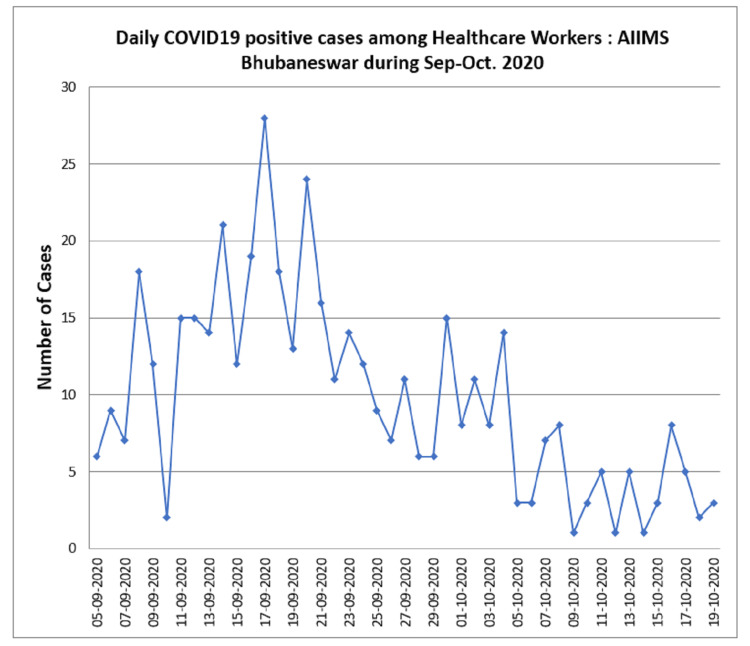
COVID-19 daily positive cases among healthcare workers during September-October 2020 COVID-19, coronavirus disease 2019

We noticed an increasing number of HCWs getting infected with SARS-CoV-2 infection in early September 2020 at our hospital, which was negatively impacting the healthcare services we had to provide (Figure 2). After carefully assessing the published information on ivermectin, we decided to investigate the role of ivermectin prophylaxis in the prevention of COVID-19 among HCWs following one month of administration in our hospital.

We observed that the HCWs who took ivermectin chemoprophylaxis had an 83% lower risk of contracting COVID-19 in the following month compared to those who did not receive the drug. Based on its long history of clinical use, favorable safety profile, and emerging evidence from the in vitro study, observational study, and open-label RCT, ivermectin was used as a prophylactic agent for COVID-19 infection in our hospital for HCWs and has shown promising results. The adverse effects reported by the subjects were few and fit into the safety profile of this drug.

Ivermectin is a widely available anti-parasitic drug and has been included in the WHO list of essential medicines. The safety of the drug has been established by its large-scale use in the last four decades for various indications such as onchocerciasis, scabies, head lice, and other parasitic infestations such as ascariasis and trichuriasis [12]. Ivermectin has been reported to inhibit the interaction between importin (IMP) α/β1 heterodimer integrase protein, which helps in the nuclear import and propagation of infection by RNA viruses. It exerts its antiviral activity against a variety of RNA viruses, including West Nile virus, influenza virus, and dengue virus [13]. An in vitro study by Caly et al. reported a nearly 5000-fold reduction in the SARS-CoV-2 viral RNA with the use of ivermectin [5]. However, a simulation study has suggested that despite a high lung:plasma concentration ratio, ivermectin would not achieve the required inhibitory concentration in the lungs after a single oral administration at the approved dose and may necessitate much higher doses [14]. Nevertheless, clinical studies have shown that the addition of ivermectin at doses ranging from 150 to 200 μg/kg body weight led to lower mortality and greater clinical improvement in COVID-19 patients. A recent meta-analysis explored its therapeutic potential in COVID-19 patients and reported a significant reduction in all-cause mortality with a pooled odds ratio of 0.53 (95% CI, 0.29-0.96, p=0.04) with the addition of ivermectin as compared to standard therapy [15]. A recent retrospective cohort study by Rajter et al. also demonstrated that ivermectin lowered mortality during treatment of COVID-19 [16].

A randomized open-label clinical trial carried out by Shouman in Egypt showed that prophylactic ivermectin therapy at an average dose of 300 μg/kg body weight in primary contacts of COVID-19 patients led to significantly lower infections (7.4%) compared to controls (58.4%) [7]. The half-life of the drug is 12-36 hours following oral administration, and it undergoes hepatic metabolism and is eliminated primarily through the faecal route over 12 days, with less than 1% being eliminated through the renal route. The active metabolites persist in the body for three days [12]. The dose regimen chosen for prophylaxis in our study was thus based on these pharmacokinetic parameters, and the fact that dosage chosen in the clinical trial by Shouman was associated with high clinical efficacy and low rate of adverse events [7]. In our study, we also found that females had a lower risk of SARS-CoV-2 infection compared to males. The previous research from India also had similar findings [17-18].

The strengths of our study are the large sample size, minimal loss to follow-up, and the establishment of temporality. The ideal study design to answer our research question would be a randomized controlled clinical trial. However, due to ethical reasons, we could not choose this design. HCWs who took ivermectin may somehow differ from the HCWs who did not prefer to take the prophylaxis in their behaviour. However, we had a strong institutional policy in place related to COVID-19-appropriate behavior in the workplace, which may have avoided the possible bias. The major limitation is that we only tested HCWs who either developed symptoms or who were direct or high-risk contacts of positive patients. This was done in keeping with the Government strategy for COVID-19 testing in India. However, this precludes us from including the HCWs who may have been asymptomatic or mildly symptomatic and chose not to get tested.

We believe that ivermectin is a low-cost prophylaxis (one 12-mg tablet cost us 8 INR/0.1 USD) that can easily be used in many settings to reduce the burden of the disease. Further research is required to guide the frequency of chemoprevention, acceptability, and cost-effectiveness in the community setting.

## Conclusions

Two doses (300 μg/kg/dose in a gap of 72 hours) of ivermectin chemoprophylaxis reduced COVID-19 infection by 83% among HCWs for one month. Ivermectin is a safe and effective strategy to prevent COVID-19, in the containment of pandemic alongside vaccine. Further research is required to guide the frequency of chemoprevention, acceptability, and cost-effectiveness in the community setting.
